# Preparation and Co‐Culture of iPSC‐Derived Dopaminergic Neurons and Astrocytes

**DOI:** 10.1002/cpcb.98

**Published:** 2019-10-04

**Authors:** Aurelie de Rus Jacquet

**Affiliations:** ^1^ Howard Hughes Medical Institute Janelia Research Campus Ashburn Virginia

**Keywords:** astrocytes, co‐culture, dopaminergic neurons, iPSC differentiation, non‐cell‐autonomous, Parkinson's disease

## Abstract

Induced pluripotent stem cell (iPSC)‐based models are powerful tools to study neurodegenerative diseases such as Parkinson's disease. The differentiation of patient‐derived neurons and astrocytes allows investigation of the molecular mechanisms responsible for disease onset and development. In particular, these two cell types can be mono‐ or co‐cultured to study the influence of cell‐autonomous and non‐cell‐autonomous contributors to neurodegenerative diseases. We developed a streamlined procedure to produce high‐quality/high‐purity cultures of dopaminergic neurons and astrocytes that originate from the same population of midbrain floor‐plate progenitors. This unit describes differentiation, quality control, culture parameters, and troubleshooting tips to ensure the highest quality and reproducibility of research results. © 2019 The Authors.

**Basic Protocol 1**: Differentiation of iPSCs into midbrain‐patterned neural progenitor cells

**Support Protocol**: Quality control of neural progenitor cells

**Basic Protocol 2**: Differentiation of neural progenitor cells into astrocytes

**Basic Protocol 3**: Differentiation of neural progenitor cells into dopaminergic neurons

**Basic Protocol 4**: Co‐culture of iPSC‐derived neurons and astrocytes

## INTRODUCTION

The study of neurodegenerative diseases such as Parkinson's disease has long been hampered by the lack of models that accurately reproduce human brain cells in vitro. However, in recent years, major technical advances enabled efficient differentiation of induced pluripotent stem cells (iPSCs) into different cell types, including dopaminergic neurons and astrocytes (Emdad, D'Souza, Kothari, Qadeer, & Germano, 2012; Kriks et al., 2011; TCW et al., 2017). These iPSC‐differentiated cells carry the same genetic signature as the donor and allow researchers to interrogate the causes of neurodegenerative diseases. This unit describes several protocols to differentiate human iPSCs into midbrain‐patterned neural progenitor cells (NPCs), astrocytes, and neurons. Importantly, it allows production of dopaminergic neurons and astrocytes originating from the same midbrain‐patterned NPC population. Astrocytes possess core properties as well as specialized functions determined by their regional identity (Chai et al., 2017; Yeh, Lee, Gianino, & Gutmann, 2009), and this specialization may play a role in the development and progression of neurodegenerative diseases (Clarke et al., 2018; Kostuk, Cai, & Iacovitti, 2019). Therefore, the ability to produce astrocytes and neurons originating from the same population of midbrain‐patterned NPCs is a key advantage for studying neurodegenerative diseases such as Parkinson's disease. The interaction and chemical communication between neurons and astrocytes are critical to maintaining neuronal health, and dysfunctional astrocytes have been shown to induce neurodegeneration (Di Giorgio, Boulting, Bobrowicz, & Eggan, 2008; Tyzack et al., 2017). Several cell culture systems allow study of astrocyte‐neuron communication, and this unit describes establishment and maintenance of a co‐culture of dopaminergic neurons and astrocytes, with four detailed protocols and one support protocol for differentiating iPSCs into midbrain‐patterned NPCs (Basic Protocol 1) and performing quality control of these cells (Support Protocol), differentiating NPCs into astrocytes (Basic Protocol 2) and dopaminergic neurons (Basic Protocol 3), and co‐culturing these dopaminergic neurons and astrocytes (Basic Protocol 4).

## STRATEGIC PLANNING

**Figure 1:**
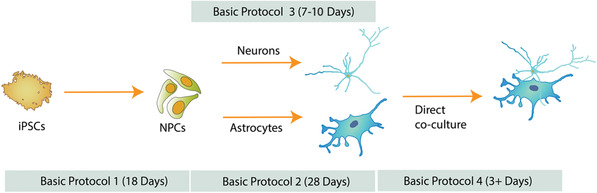
Overview of production of iPSC‐derived NPCs, astrocytes, and dopaminergic neurons and direct co‐culture of the dopaminergic neurons and astrocytes.

*NOTE*: All culture incubations are at 37°C unless otherwise specified.

*NOTE*: Cells should be handled according to BSL‐2 regulations. Cells and reagents must be maintained under sterile conditions at all times, and biohazard waste needs to be disposed of accordingly.

The timing of differentiation varies depending on the cell type of interest (Fig. 1). As a consequence, it is important to anticipate and time the production of each cell type according to the desired experiment. NPCs and astrocytes can be produced in large quantities and cryopreserved to make stocks readily available to users. Successful cryopreservation of iPSC‐derived neurons has also been described (Wakeman et al., 2017); however, we usually prepare fresh neurons 7 to 10 days before a co‐culture experiment.

## Basic Protocol 1 DIFFERENTIATION OF iPSCs INTO MIDBRAIN‐PATTERNED NEURAL PROGENITOR CELLS

NPCs have the ability to differentiate into neural cell types including neurons and astrocytes (Tcw et al., 2017). When these NPCs are patterned toward a specific regional identity, they can produce neurons specific to that brain region (Kirkeby et al., 2012; Kriks et al., 2011). The production of iPSC‐derived dopaminergic neurons described by Kriks et al. (2011) is a two‐phase protocol, with initial midbrain floor‐plate patterning of neural progenitors, followed by differentiation of these progenitors into neurons (Kriks et al., 2011). The protocol described here is an adaptation of that original protocol: it adheres to Kriks’ initial 11 days of differentiation and then differs by establishing a population of self‐renewing midbrain‐patterned NPCs. The advantage of this method is the possibility to produce large numbers of NPCs that can be frozen and stored. These NPCs can be returned to culture and readily used to produce astrocytes (Basic Protocol 2) and neurons (Basic Protocol 3), alleviating the need for maintaining iPSCs and reducing the risk of variability between downstream differentiations.

**Figure 2:**
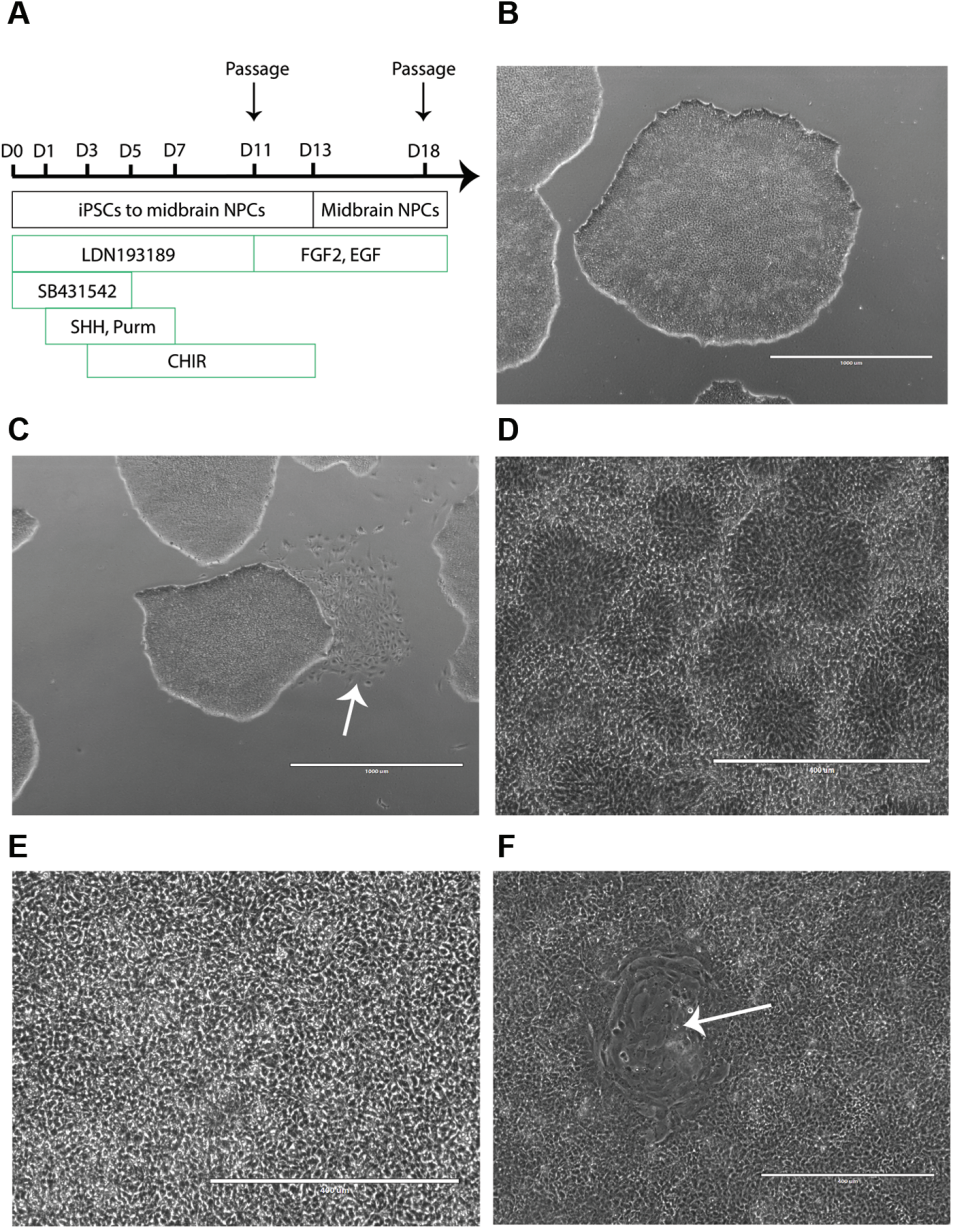
Timeline of differentiation of iPSCs into midbrain‐patterned NPCs (**A**). Healthy iPSC colony with tight edges (**B**). Areas of spontaneous differentiation that need to be manually removed before differentiation (white arrow) (**C**). Representative images of healthy NPC cultures at 4× (**D**) and 10× (**E**) magnification. Occasionally, contaminants (such as large, flat cells; white arrow) propagate in the culture (**F**). These contaminants tend to develop when the NPC culture is not plated at a density of at least 7.4 × 10^5^ cells/cm^2^.

To initiate differentiation, iPSCs are plated as a monolayer and subsequently neuralized via dual SMAD inhibition (Fig. 2A). After 11 days in culture, the cells are passaged; cultured for an additional 7 days; and subsequently frozen, sub‐cultured, or differentiated into regionalized astrocytes (Basic Protocol 2) and neurons (Basic Protocol 3). Newly produced NPCs need to undergo quality control (Support Protocol) to ensure expression of region‐specific marker genes.

### Materials

Confluent iPSCs in growth medium in 10‐cm dish
mTeSR1 or mTeSR Plus medium (Stemcell Technologies, cat. no. 85870 or 05825; store in aliquots at −20°C)
Accumax (Innovative Cell Technologies, cat. no. AM105‐500; store in aliquots at −20°C)
Dulbecco's phosphate‐buffered saline (DPBS; Thermo Fisher, cat. no. 14190250)
ROCK inhibitor (Y‐27632, Stemcell Technologies, cat. no. 72304)
Serum‐free medium (SRM; see recipe), 37°C
LDN193189 hydrochloride (Sigma, cat. no. SML0559)
SB431542 (Stemcell Technologies, cat. no. 72234)
SHH(C25II), recombinant mouse protein (SHH; R&D Systems, cat. no. 464‐SH‐200)
Purmorphamine (Millipore, cat. no. 540223)
CHIR99021 (Stemcell Technologies, cat. no. 72054)
N2 medium (see recipe), 37°C
Complete neurobasal medium (see recipe), 37°C
NPC medium (see recipe), 37°C

Light microscope
15‐ml conical centrifuge tubes
Standard tabletop centrifuge
Hemocytometer
Geltrex‐coated 6‐well plates (see recipe)

### Day ‐1

1 Observe confluent iPSCs in growth medium in a 10‐cm dish under a light microscope to monitor cell health, ensuring that colonies with tight edges and a dense center are present (Fig. 2B). Under sterile conditions, manually remove areas of spontaneous differentiation using tip of a pipet tip (Fig. 2C).
  We suggest growing at least one 10‐cm dish of iPSCs. The iPSC culture is ready for plating when it is confluent and consists of large colonies without spontaneous differentiation.
2 Pre‐warm aliquots of mTeSR1 or mTeSR Plus medium and Accumax to 37°C. Equilibrate DPBS at room temperature.
  mTeSR1 and mTeSR1 Plus media are both excellent choices for growing iPSCs. However, mTeSR1 Plus medium allows feeding of iPSCs every other day instead of daily, and doubling the volume of medium allows one to skip feeding on the weekend.
3 Prepare 10 ml pre‐warmed mTeSR1 medium supplemented with 10 µM ROCK inhibitor.
  ROCK inhibitor promotes iPSC survival when iPSCs are plated as single cells.
  The volume of mTeSR1 medium supplemented with ROCK inhibitor may need to be adjusted depending on the number of cells harvested and the number of wells for cell plating.
4 Remove growth medium from the iPSCs from step 1 and wash cells once with 5 ml DPBS. Add 3 ml pre‐warmed Accumax.
5 Incubate cells at 37°C until they become rounded and detach from the dish.
  Monitor cells after a 3‐min incubation. A longer incubation time may be necessary, but no longer than 10 min. A longer incubation time may reduce the viability of the cells.
6 Gently triturate cells to prepare a single‐cell suspension. Transfer cell suspension to a 15‐ml conical centrifuge tube.
  To prevent a reduction in cell viability, do not over‐triturate.
7 Wash the 10‐cm dish with 8 ml mTeSR1 medium to collect as many cells as possible and transfer to 15‐ml tube containing the cell suspension.
8 Centrifuge 3 min at 300 × *g*. Aspirate supernatant and resuspend cells in 3 ml mTeSR1 medium supplemented with 10 µM ROCK inhibitor (see step 3).
9 Count cells with a hemocytometer and plate at a density of 1.9 × 10^6^ cells/2 ml/well in a Geltrex‐coated 6‐well plate.
  The optimum cell density needs to be empirically determined for each iPSC line. The optimum cell density is defined as the density that will result in a confluent monolayer of iPSCs the next day, a healthy culture until Day 11 of the protocol, and ultimately successful generation of midbrain‐patterned NPCs. This may require more than one 10‐cm dish, in which case the volumes detailed above would need to be adjusted.

### Day 0

10 The next day, wash cells with 2 ml DPBS per well to remove dead cells and debris.
  A substantial proportion of cells do not survive after plating. This is normal, but it is critical to ensure that the culture consists of a monolayer of healthy cells after plating. If the cells do not survive, prepare fresh stocks of ROCK inhibitor. Alternatively, replace the ROCK inhibitor with RevitaCell Supplement (Thermo Fisher, cat. no. A2644501).
11 Monitor cell viability under a light microscope, ensuring a confluent monolayer of iPSCs. If there are areas of the plate without cells, let culture grow for one more day in fresh mTeSR1 medium without ROCK inhibitor. If the monolayer is ready for differentiation, add 2 ml SRM freshly supplemented with 100 nM LDN193189 hydrochloride and 10 µM SB431542.

### Day 1

12 Aspirate Day 0 medium and add SRM freshly supplemented with 100 nM LDN193189, 10 µM SB431542, 100 ng/ml SHH, and 2 µM purmorphamine.

### Day 2

13 Aspirate Day 1 medium and add SRM freshly supplemented with 100 nM LDN193189, 10 µM SB431542, 100 ng/ml SHH, and 2 µM purmorphamine.

### Day 3

14 Aspirate Day 2 medium and add SRM medium freshly supplemented with 100 nM LDN193189, 10 µM SB431542, 100 ng/ml SHH, 2 µM purmorphamine, and 3 µM CHIR99021.

### Day 5

15 Combine SRM and N2 medium at a 3:1 ratio. Supplement with 100 nM LDN193189, 100 ng/ml SHH, 2 µM purmorphamine, and 3 µM CHIR99021.
  For example, to make 10 ml Day 5 medium, combine 7.5 ml SRM and 2.5 ml N2 medium. Add the supplements, and the complete medium is ready.
16 Aspirate medium from the cells and feed cells with the medium prepared above.

### Day 7

17 Combine SRM and N2 medium at a 1:1 ratio. Supplement with 100 nM LDN193189 and 3 µM CHIR99021.
  For example, to make 10 ml Day 7 medium, combine 5 ml SRM and 5 ml N2 medium. Add the supplements, and the complete medium is ready.
18 Aspirate medium from the cells and feed cells with the medium prepared above.

### Day 9

19 Combine SRM and N2 medium at a 1:3 ratio. Supplement with 100 nM LDN193189 and 3 µM CHIR99021.
  For example, to make 10 ml Day 9 medium, combine 2.5 ml SRM and 7.5 ml N2 medium. Add the supplements, and the complete medium is ready.
20 Aspirate medium from the cells and feed cells with the medium prepared above.

### Day 11

21 Passage cells as follows:
  - Wash cells once with 2 ml DPBS per well and add 1 ml pre‐warmed Accumax per well.
  - Incubate at 37°C for 3 min. After 3 min, monitor cells under a light microscope.
    If the cells are rounded and detached from the plate, they are ready to harvest. If not, incubate 2 to 3 min longer in the incubator. Do not over‐incubate because it may reduce the viability of the cells.
  - Transfer cell suspension to a 15‐ml conical centrifuge tube and add 5 ml complete neurobasal medium. Wash wells with 1 ml complete neurobasal medium to collect the remaining cells and transfer to 15‐ml tube containing the cell suspension.
  - Centrifuge cell suspension for 3 min at 300 × *g*. Aspirate supernatant and resuspend pellet in NPC medium freshly supplemented with 3 µM CHIR99021 and 10 µM ROCK inhibitor.
    The resuspension volume varies depending on the number of wells collected. It is recommended to resuspend in 1 ml per collected well.
  - Count cells and plate at a density of 7 × 10^6^ cells/2 ml/well in a Geltrex‐coated 6‐well plate. Label plate with the passage of the iPSCs at the time of plating and the NPC passage number.
    For example, if the iPSCs were passage 20 when plated at Day ‐1, label the plate P_20_N_0_. This step is passage 0 of the NPCs. The NPCs are not fully patterned yet and require an additional 2 days of patterning with CHIR99021.

### Day 12

22 The next day, monitor cell viability under a light microscope.
  A significant number of cells will be dead, but the culture should consist of a very confluent monolayer.
23 Aspirate medium and wash cells once with 2 ml DPBS to remove the dead cells and debris.
24 Feed cells with NPC medium freshly supplemented with 3 µM CHIR99021.

### Days 13 to 16

25 Each day, aspirate medium and feed cells with NPC medium.

### Day 18

26 Passage cells on Day 18 and then every 7 days at 7 × 10^6^ cells/well in NPC medium freshly supplemented with 10 µM ROCK inhibitor in a Geltrex‐coated 6‐well plate.
  The NPCs form a dense monolayer that should be free of contaminants (Fig. 2D–F).

## QUALITY CONTROL OF NEURAL PROGENITOR CELLS

**Figure 3:**
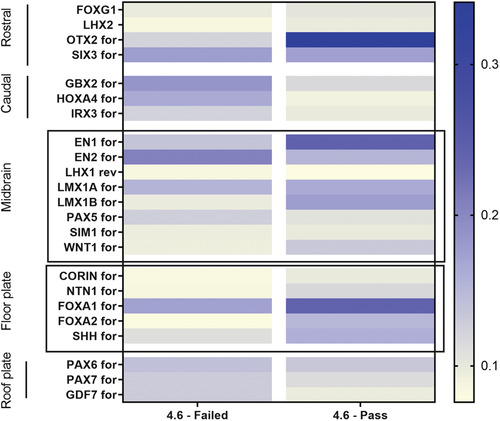
Example of NPC quality control. Two NPC lines were generated from the same 4.6 iPSC line. However, one NPC line failed quality control, whereas the other passed. The NPC line that failed the quality control shows lower expression levels of floor‐plate and midbrain gene markers. This same line also shows greater levels of roof‐plate and caudal marker genes, which are not desirable when producing dopaminergic neurons.

This protocol describes quality control of the NPCs obtained in Basic Protocol 1. It is very important to perform and document this quality control to ensure that the NPCs express gene markers of a midbrain and floor‐plate fate (Fig. 3). It is suggested to perform this quality control once, early after differentiation, such as at passage 1 or 2. If the NPCs do not express satisfactory levels of marker genes, it is likely that they will not produce desirable yields of dopaminergic neurons (Basic Protocol 3). In such a case, new NPCs will need to be produced. To confirm NPC identity (rather than their brain‐region patterning), additional techniques such as immunofluorescence can be used to check for expression of NPC markers (not described here; Cheng et al., 2015).

### Materials

NPCs (see Basic Protocol 1)
NPC medium (see recipe), 37°C
DPBS (Thermo Fisher, cat. no. 14190250)
TRIzol reagent (Thermo Fisher, cat. no. 15596026)
RNA purification kit (Qiagen, cat. no. 74104)
cDNA synthesis kit (Thermo Fisher Scientific, cat. no. K1622)
qPCR kit (Roche, cat. no. 07959583001)

Geltrex‐coated 12‐well plates (see recipe)
1.5‐ml Eppendorf tubes

Additional reagents and equipment for RNA purification, cDNA synthesis, and qPCR (see manufacturer's guidance)

*CAUTION*: TRIzol is a toxic reagent. To prevent inhalation of vapors, always work in a chemical hood. Use appropriate personal protective equipment, such as gloves and a laboratory coat. Set up a waste container to collect TRIzol‐contaminated material (e.g., pipet tips, tubes).

1 Plate NPCs into individual wells in a Geltrex‐coated 12‐well plate at a density of 3 × 10^6^ cells/well. Feed NPCs with 1 ml NPC medium daily for 48 hr.
2 After 48 hr, transfer plate to a chemical hood. Wash cells once with 1 ml DPBS and add 500 µl TRIzol reagent.
3 After 5 min, triturate contents of the well and collect TRIzol extraction into a 1.5‐ml Eppendorf tube. Process sample for RNA extraction and cDNA synthesis immediately using RNA purification and cDNA synthesis kits, respectively, or store at −80°C.
4 Perform qPCR using a qPCR kit and NPC gene panel available in Table 1 and calculate 1/delta Ct value for each gene.
  The NPC differentiation protocol produces progenitors with a floor‐plate and midbrain fate. Therefore, genes characteristic of the floor plate and midbrain should be more highly expressed than genes of the roof plate and caudal regions.

**Table 1:** Primer Sequences for Quality Control of iPSC‐Derived NPCs by qPCR

Gene name^a^	Forward primer	Reverse primer
FOXG1	TGGCCCATGTCGCCCTTCCT	GCCGACGTGGTGCCGTTGTA
LHX2	GGGCGACCACTTCGGCATGAA	CGTCGGCATGGTTGAAGTGTGC
OTX2	ACAAGTGGCCAATTCACTCC	GAGGTGGACAAGGGATCTGA
SIX3	ACCGGCCTCACTCCCACACA	CGCTCGGTCCAATGGCCTGG
GBX2	GTTCCCGCCGTCGCTGATGAT	GCCGGTGTAGACGAAATGGCCG
HOXA4	ACGCTCTGTTTGTCTGAGCGCC	AGAGGCCGAGGCCGAATTGGA
IRX3	GGCTTGCGCCCCGTAGAAATGT	AGGAGCCAGGTCAGGTCCGAAC
EN1	CGTGGCTTACTCCCCATTTA	TCTCGCTGTCTCTCCCTCTC
EN2	CCTCCTGCTCCTCCTTTCTT	GACGCAGACGATGTATGCAC
LHX1	AGGTGAAACACTTTGCTCCG	CTCCAGGGAAGGCAAACTCT
LMX1A	CGCATCGTTTCTTCTCCTCT	CAGACAGACTTGGGGCTCAC
LMX1B	CTTAACCAGCCTCAGCGACT	TCAGGAGGCGAAGTAGGAAC
PAX5	CCCCATTGTGACAGGCCGTGAC	TCAGCGTCGGTGCTGAGTAGCT
SIM1	AAAGGGGGCCAAATCCCGGC	TCCGCCCCACTGGCTGTCAT
WNT1	GAGCCACGAGTTTGGATGTT	TGCAGGGAGAAAGGAGAGAA
CORIN	CATATCTCCATCGCCTCAGTTG	GGCAGGAGTCCATGACTGT
NTN1	GCATGCAGGTTGCAGTTACA	GCTGCAAGCCCTTCCACTA
FOXA1	GGGCAGGGTGGCTCCAGGAT	TGCTGACCGGGACGGAGGAG
FOXA2	CCGTTCTCCATCAACAACCT	GGGGTAGTGCATCACCTGTT
SHH	CCAATTACAACCCCGACATC	AGTTTCACTCCTGGCCACTG
PAX6	TGGTATTCTCTCCCCCTCCT	TAAGGATGTTGAACGGGCAG
PAX7	CTTCAGTGGGAGGTCAGGTT	CAAACACAGCATCGACGG
GDF7	GACGCTGCTCAACTCCATGGCA	TTGGCGGCGTCGATGTAGAGGA

a This list of primers was published by Kirkeby and colleagues and is used to establish a quality‐control gene panel (Kirkeby et al., 2012).

## Basic Protocol 2 DIFFERENTIATION OF NEURAL PROGENITOR CELLS INTO ASTROCYTES

**Figure 4:**
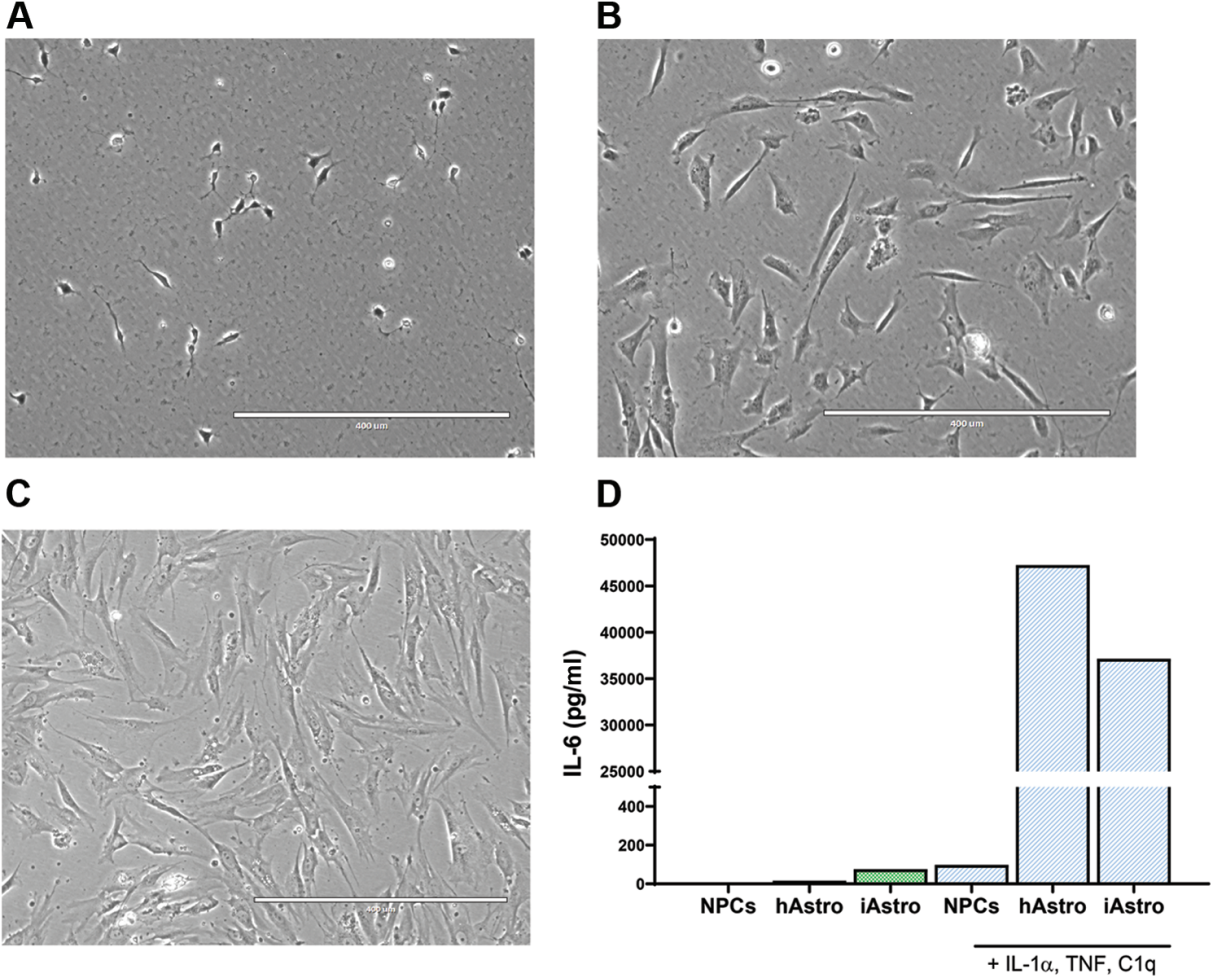
Representative images of NPCs during astrocyte differentiation at Day 0, 1 day after plating NPCs (**A**); Day 7, 1 day after plating A_0_; (**B**) and Day 21, 1 day after plating A_3_. (**C**). Successfully differentiated astrocytes respond to stimulation by IL‐1α (3 ng/ml), TNF (30 ng/ml), and C1q (400 ng/ml) by secreting IL‐6 (**D**). The morphology of the cells changes from small and round to large and flat. The morphology of the astrocytes does not usually significantly change after Day 21 and can slightly vary depending on the NPC line; for example, some astrocyte lines may look more elongated than others. hAstro: human midbrain astrocytes (ScienCell, cat. no. 1850); iAstro: iPSC‐derived astrocytes.

Differentiation of astrocytes can be achieved by different methods, using either iPSCs or neuralized progenitors as starting material (Chandrasekaran, Avci, Leist, Kobolak, & Dinnyes, 2016). TCW and colleagues described production of astrocytes from forebrain NPCs using a commercial medium produced by ScienCell Research Laboratories (TCW et al., 2017). This method has many advantages: it requires a single type of medium, involves little hands‐on time, and produces functioning astrocytes in a relatively short period of time (28 days). However, it requires supplementation of medium with 2% fetal bovine serum (FBS), which may affect downstream applications. Basic Protocol 2 describes production of astrocytes from a population of midbrain‐patterned NPCs (Basic Protocol 1) using ScienCell astrocyte medium. To initiate differentiation, NPCs are plated at low density, continuously maintained in astrocyte differentiation medium, and passaged every 7 days until Day 28. The growth rate of different NPC lines may vary, especially in the first 14 days of differentiation. Therefore, the timing of cell passaging should be adjusted according to the NPC line of interest. Cell morphology is a visual indicator of differentiation and progressively changes, from small NPCs to large, flat astrocytes (Fig. 4A‐C).

### Materials

NPCs (see Basic Protocol 1)
DPBS (Thermo Fisher, cat. no. 14190250)
Accumax (Innovative Cell Technologies, cat. no. AM105‐500; store in aliquots at −20°C), 37°C
Complete neurobasal medium (see recipe), 37°C
NPC medium (see recipe), 37°C
ROCK inhibitor (Y‐27632; Stemcell Technologies, cat. no. 72304)
Astrocyte medium (ScienCell Research Laboratories, cat. no. 1801), 37°C
Astrocyte freezing medium: astrocyte medium supplemented with 10% (v/v) dimethylsulfoxide (DMSO)

Geltrex‐coated 6‐well plates and 10‐ and 15‐cm dishes (see recipe)
15‐ and 50‐ml conical centrifuge tubes
Standard tabletop centrifuge
Hemocytometer

### Day 1

1 Maintain NPCs in one well of a Geltrex‐coated 6‐well plate (see Basic Protocol 1, step 26). Seven days after the previous passage, split cells:
  - Coat one well of a 6‐well plate with Geltrex (see Reagents and Solutions) ≥1 hr prior to plating.
  - Wash NPCs once with 2 ml DPBS. Aspirate DPBS and add 1 ml pre‐warmed Accumax per well. Incubate at 37°C until cells detach (3 to 6 min).
  - Transfer cell suspension to a 15‐ml conical centrifuge tube and add 5 ml complete neurobasal medium.
  - Centrifuge cell suspension for 3 min at 300 × *g*. Aspirate supernatant and resuspend pellet in 2 ml NPC medium freshly supplemented with 10 µM ROCK inhibitor. Count cells with a hemocytometer and plate at a density of 15,000 cells/cm^2^ in one well of Geltrex‐coated 6‐well plate. To keep track of the history of the cells, label plate with the passage number of the iPSCs, followed by the passage number of the NPCs and the passage number of the astrocytes.
    For example, the label P_30_N_4_A_0_ means that the iPSCs were at passage 30 when they were differentiated into NPCs. These NPCs were at passage 4 when they were plated to differentiate into astrocytes. The astrocytes are labeled A_0_ in their first week of differentiation, resulting in the final label P_30_N_4_A_0_.

### Day 0

2 Wash ‐cells once with 2 ml DPBS and add 2 ml astrocyte medium.
  From Day 0 until complete differentiation, the cells will be maintained in only ScienCell astrocyte medium.

### Days 2, 4, and 6

3 Change medium on Day 2 and then every other day until Day 7.

### Day 7

4 Passage cells as in step 1, with slight modifications:
  - Coat all wells of 6‐well plate with Geltrex.
  - Centrifuge cell suspension in astrocyte medium instead of complete neurobasal medium.
  - Plate 15,000 cells/cm^2^ in up to six wells of Geltrex‐coated 6‐well plate.
    After this first passage, the astrocytes are labeled A_1_.
    Continuing from the previous example (see step 1), the culture would now be labeled P_30_N_4_A_1_.

### Day 9

5 Change medium on Day 9 and then every other day until Day 14.

### Day 14

6 Passage cells as in step 4, with slight modifications:
  - Coat three 10‐cm dishes with Geltrex.
  - Plate dishes at 15,000 cells/cm^2^.
    After this second passage, the astrocytes are labeled A_2_.
    Continuing from the previous example (see step 4), the culture would now be labeled P_30_N_4_A_2_.

### Day 16

7 Change medium on Day 16 and then every other day until Day 21.

### Day 21

8 Passage cells as in step 4, with slight modifications:
  - Coat three 15‐cm dishes with Geltrex.
  - Add 3 ml pre‐warmed Accumax to each dish.
  - Transfer cell suspension to a 50‐ml conical centrifuge tube and add 15 ml astrocyte medium.
  - Resuspend pellet in 5 ml astrocyte medium. Plate dishes at 15,000 cells/cm^2^.
    After this third passage, the astrocytes are labeled A_3_.
    Continuing from the previous example (see step 6), the culture would now be labeled P_30_N_4_A_3_.

### Day 23

9 Change medium on Day 23 and then every other day until Day 28.

### Day 28

10 Repeat step 4, with slight modifications:
  - If astrocytes will be plated for experiments, prepare Geltrex‐coated plates as necessary.
  - Add 5 ml pre‐warmed Accumax to each 15‐cm dish.
  - Transfer cell suspension to a 50‐ml conical centrifuge tube and add 20 ml astrocyte medium.
  - Resuspend pellet in 10 ml astrocyte medium. Plate at desired density for experiments in Geltrex‐coated plates.
    After this fourth passage, the astrocytes are labeled A_4_.
  - Freeze remaining cells at the desired density in astrocyte freezing medium, including ≥30% additional cells to account for cell loss during handling and thawing.
    Day 28 is the final astrocyte differentiation stage. The astrocytes can be frozen and/or plated for experiments.
    Depending on the cell line, the rate of astrocyte growth can decrease after the final differentiation stage.
    Appropriate quality control of the astrocytes (not described here) should be performed. This includes at least immunofluorescence staining of astrocytic markers (e.g., GFAP, CD44, vimentin; TCW et al., 2017; di Domenico et al., 2019) and a functional assay such as an IL‐6 cytokine secretion assay (Fig. 4D).

## Basic Protocol 3 DIFFERENTIATION OF NEURAL PROGENITOR CELLS INTO DOPAMINERGIC NEURONS

**Figure 5:**
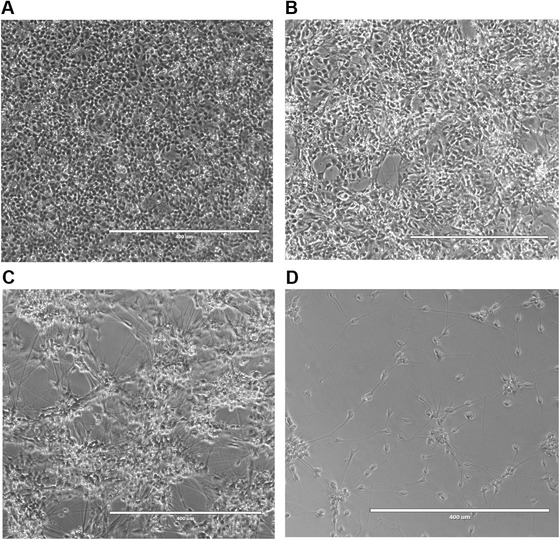
Representative images of iPSC‐derived dopaminergic neurons at Day 0 (**A**), Day 3 (**B**), and Day 7 (**C**) and after MACS isolation of CD133^‐^ dopaminergic neurons (**D**). During differentiation, a large number of NPCs progressively die, and the surviving cells either differentiate into neurons or remain undifferentiated. The undifferentiated CD133^+^ NPCs are depleted by MACS, resulting in a highly enriched population of neurons.

The following protocol describes differentiation of regionalized NPCs (Basic Protocol 1) into dopaminergic neurons. For differentiation, NPCs are plated at a high density and subsequently incubated in dopaminergic neuron differentiation medium (Fig. 5A‐C). Between Day 7 and Day 10, most cells extend long neurites (Fig. 5C), whereas others fail to differentiate. The culture is then depleted of these undifferentiated CD133^+^ NPCs by magnetic‐activated cell sorting (MACS), resulting in a highly enriched neuronal culture ready to be plated for experiments (Fig. 5D).

### Materials

NPCs (see Basic Protocol 1)
Geltrex‐coated 12‐well and other plates (see recipe)
DPBS (Thermo Fisher, cat. no. 14190250)
Accumax (Innovative Cell Technologies, cat. no. AM105‐500; store in aliquots at −20°C), 37°C
Complete neurobasal medium (see recipe), 37°C
NPC medium (see recipe), 37°C
ROCK inhibitor (Y‐27632; Stemcell Technologies, cat. no. 72304)
Dopaminergic neuron differentiation medium (see recipe), 37°C
MACS buffer (see recipe)
APC mouse anti‐human CD133 (BD Biosciences, cat. no. 566596)
APC‐conjugated magnetic beads (Anti‐APC MicroBeads, Miltenyi, cat. no. 130‐090‐855)
Astrocytes (see Basic Protocols 2 and 4; optional)

15‐ml conical centrifuge tubes
Standard tabletop centrifuge, room temperature or 4°C
Hemocytometer
Strainer tube (round‐bottom polystyrene test tube with cell‐strainer snap cap, Corning, cat. no. 352235)
1.5‐ml Eppendorf tubes
LD column (Miltenyi, cat. no. 130‐042‐901)
Multistand (MACS MultiStand, Miltenyi, cat. no. 130‐042‐303)
Separator (MidiMACS Separator, Miltenyi, cat. no. 130‐042‐302)

1 Maintain NPCs in one well of a Geltrex‐coated 6‐well plate (see Basic Protocol 1, step 26). Seven days after the previous passage, split cells:
  - Coat one well of a 12‐well plate with Geltrex (see Reagents and Solutions) ≥1 hr prior to plating.
  - Wash NPCs once with 2 ml DPBS and add 1 ml pre‐warmed Accumax. Incubate at 37°C until cells detach (3 to 6 min).
  - Transfer cell suspension to a 15‐ml conical centrifuge tube and add 5 ml complete neurobasal medium.
  - Centrifuge cell suspension for 3 min at 300 × *g*. Aspirate supernatant and resuspend pellet in 2 ml NPC medium freshly supplemented with 10 µM ROCK inhibitor. Count cells with a hemocytometer and plate 3 × 10^6^ cells in 1 ml in one well of Geltrex‐coated 12‐well plate.
2 The next day, wash cells once with 1 ml DPBS and add 1 ml dopaminergic neuron differentiation medium. Feed cells every other day with dopaminergic neuron differentiation medium.
3 After 7 to 10 days, deplete neuronal culture of undifferentiated NPCs by MACS and plate cells for experiments:
  - Coat plates with Geltrex (see Reagents and Solutions).
    The type of plate depends on the purpose of the experiment. For example, 96‐ or 48‐well plates with optical bottoms or coverslips are useful for imaging experiments. For biochemical experiments, it is recommended to use larger plates, such as 12‐ or 6‐well plates.
  - Wash cells once with 1 ml DPBS and add 0.5 ml pre‐warmed Accumax. Incubate at 37°C for 10 min.
  - Transfer cell suspension to a 15‐ml conical centrifuge tube and add 5 ml complete neurobasal medium. Centrifuge cell suspension for 3 min at 300 × *g*. Aspirate supernatant and resuspend pellet in 500 µl complete neurobasal medium.
  - Prepare a strainer tube by passing 300 µl complete neurobasal medium through it. Filter cell suspension to remove large aggregates and collect single cells. Wash membrane with 400 µl complete neurobasal medium. Discard filter, transfer cell suspension to a 1.5‐ml Eppendorf tube, and centrifuge 3 min at 300 × *g*.
  - Resuspend pellet in 1 ml MACS buffer and count total number of cells. Centrifuge 3 min at 300 × *g*.
  - Resuspend every 1 × 10^6^ cells in 95 µl MACS buffer supplemented with 5 µl APC mouse anti‐human CD133. Incubate at 4°C for 10 min.
  - Add 1 ml MACS buffer and centrifuge 3 min at 300 × *g*, 4°C.
  - Resuspend pellet in 80 µl MACS buffer supplemented with 20 µl APC‐conjugated magnetic beads (≤10^8^ cells per 100 µl final volume). Incubate at 4°C for 10 min.
  - To magnetically deplete undifferentiated NPCs, set up MACS multistand and separator according to the manufacturer's instructions and prepare the LD column by applying 2 ml MACS buffer to the column and discarding flow‐through. When the column is ready, set up a 1.5‐ml Eppendorf tube to collect flow‐through containing the neurons.
  - Add 400 µl MACS buffer to cell suspension to dilute it and filter through column as described in the manufacturer's instructions. Collect flow‐through in the 1.5‐ml tube.
    The flow‐through contains the unlabeled neurons. The magnetically labeled CD133^+^ NPCs are retained in the column. Do not remove the column from the magnetic field (MACS multistand and separator) until the cell sorting is completed.
  - Wash column by applying 500 µl MACS buffer and collect cells in the flow‐through in the same 1.5‐ml tube. Repeat wash once.
  - Centrifuge 1.5‐ml tube for 3 min at 300 × *g*, 4°C. Discard LD column.
  - Gently resuspend cell pellet in 0.5 ml dopaminergic neuron differentiation medium, count cells, and plate on Geltrex‐coated plates at 55,000 to 70,000 cells/well (96‐well plate) or on astrocytes (see Basic Protocol 4).
4 To avoid disturbing the neurons, replace only 50% of the dopaminergic neuron differentiation medium every 2 days until cells are ready for analysis.
  There may be occasional undifferentiated NPCs remaining in the neuronal culture after MACS sorting. Under certain culture conditions, these NPCs may divide, so treatment with cytosine β‐D‐arabinofuranoside (AraC) may be necessary to stop their propagation. The concentration and duration of AraC treatment need to be optimized for each application, but 5 µM AraC for 24 hr is a good starting point.

## Basic Protocol 4 CO‐CULTURE OF iPSC‐DERIVED NEURONS AND ASTROCYTES

Communication between neurons and astrocytes regulates a variety of processes that can be recapitulated in vitro (di Domenico et al., 2019; Hasel et al., 2017; Ioannou et al., 2019). To investigate these interactions, different culture systems can be implemented, such as direct or indirect co‐culture as well as culture in conditioned medium prepared from neurons or astrocytes. The following protocol describes the steps needed to establish a direct iPSC‐derived neuron/astrocyte co‐culture that ensures optimal viability of both neurons and astrocytes for ≥2 weeks.

### Materials

iPSC‐derived astrocytes (see Basic Protocol 2)
DPBS (Thermo Fisher, cat. no. 14190250)
Accumax (Innovative Cell Technologies, cat. no. AM105‐500; store in aliquots at −20°C), 37°C
Astrocyte medium (ScienCell Research Laboratories, cat. no. 1801), 37°C
Geltrex‐coated 96‐well plate (see recipe)
2× AraC (Sigma, cat. no. C1768) concentrated solution (i.e., 10 to 16 µM)
Complete neurobasal medium (see recipe) supplemented with 0.5% (v/v) FBS (Thermo Fisher, cat. no. A3160601), 37°C

15‐ml conical centrifuge tubes
Standard tabletop centrifuge
Hemocytometer

Additional reagents and equipment for preparation of iPSC‐derived dopaminergic neurons (see Basic Protocol 3)

*CAUTION*: AraC is an antimitotic agent and must be handled with caution.

1 Prepare iPSC‐derived astrocytes as described in Basic Protocol 2 or thaw a vial of frozen astrocytes. Culture cells until confluent.
  When astrocytes are confluent, they are ready to be passaged.
2 Wash cells once with 1 or 3 ml DPBS and incubate in 1 or 3 ml pre‐warmed Accumax at 37°C until cells detach from the plate or dish. Transfer cell suspension to a 15‐ml conical centrifuge tube and add 5 ml astrocyte medium.
  The volumes of DPBS and Accumax depend on the plate or dish used. It is recommended to use 1 ml per well of a 6‐well plate and 3 ml per 10‐cm dish.
3 Centrifuge cell suspension for 3 min at 300 × *g*. Aspirate supernatant and resuspend pellet in 1 ml astrocyte medium. Count cells with a hemocytometer and plate 9600 cells/100 µl/well in a Geltrex‐coated 96‐well plate.
  Occasionally, the astrocytes do not attach evenly to the wells and concentrate at the center or near the edges. To prevent this, first move the plate with several back‐and‐forth and left‐to‐right motions at the time of plating. Then, do not move the plate and let the cells attach for 15 to 30 min at room temperature. Transfer the plate to a cell culture incubator without perturbing the cells.
4 The next morning, supplement astrocyte medium with 5 to 8 µM AraC by adding 100 µl of 2× AraC concentrated solution (i.e., 10 to 16 µM) directly to each well (1× final).
  Treat the astrocytes with the AraC‐containing medium for 24 hr to stop cellular division. The optimal AraC concentration may vary depending on the astrocyte line.
5 After 24 hr, remove AraC‐containing medium and replace it with 200 µl fresh astrocyte medium per well.
  When removing the medium from the wells of the 96‐well plate, it is advised to use a manual pipet with a 200‐µl tip and to avoid use of an aspirator, which could lift the cells off the plate.
  The astrocytes are ready for co‐culture, and they must be used within 1 week. If the astrocytes are not used immediately, return the plate to the incubator and change the medium every 3 days.
6 Prepare iPSC‐derived dopaminergic neurons as described in Basic Protocol 3. After MACS depletion of the culture, resuspend CD133^‐^ neurons in 300 µl complete neurobasal medium supplemented with 0.5% FBS. Count cells with a hemocytometer and plate 10,000 to 13,000 neurons/200 µl/well into 96‐well plates pre‐plated with AraC‐treated, iPSC‐derived astrocytes (see step 5).
  The optimal density for neuron plating may vary depending on the neuron line and experimental needs.
7 Maintain the co‐culture in complete neurobasal medium supplemented with 0.5% FBS and only change 50% of the medium every 2 to 3 days.
  The 50% medium change will allow the nutrients to be replenished without perturbing the neurons. The co‐culture can be maintained for ≤3 weeks. Longer culture may exhibit loss of viability of the neurons and/or astrocytes.

## REAGENTS AND SOLUTIONS

### Complete neurobasal medium

Neurobasal medium (Thermo Fisher, cat. no. 21103‐049)
2% (v/v) B27 supplement (minus vitamin A; Thermo Fisher, cat. no. 12587010)
100 U/ml penicillin‐streptomycin (10,000 U/ml stock; Thermo Fisher, cat. no. 15140122)
1% (v/v) GlutaMAX (Thermo Fisher, cat. no. 35050061)
Filter sterilize using filter bottle (e.g., Millipore, cat. no. SCGVU02RE)
Store ≤3 weeks at 4°C
  The storage time is given assuming that users do not warm up the entire bottle, but instead warm up aliquots prepared from the stock medium.

### Dopaminergic neuron differentiation medium

Complete neurobasal medium (see recipe)
0.2 mM ascorbic acid (Sigma, cat. no. A92902)
0.5 mM dibutyryl cAMP (Millipore Sigma, cat. no. 28745)
10 µM DAPT (Tocris Bioscience, cat. no. 2634)
20 ng/ml recombinant human BDNF (PeproTech, cat. no. 450‐02)
20 ng/ml recombinant human GDNF (PeproTech, cat. no. 450‐10)
1 ng/ml recombinant human TGF‐β3 (R&D Systems, cat. no. 8420‐B3‐025)
Store ≤4 days at 4°C

### Geltrex‐coated plates or dishes

To aliquot Geltrex:

1. Thaw Geltrex (Fisher Scientific, cat. no. A1413302) overnight on ice at 4°C (e.g., in a small Styrofoam box filled with ice in a laboratory refrigerator).
2. The next morning, pre‐chill 1.5‐ml Eppendorf tubes, tube rack, and pipet tips.
3. Mix thawed Geltrex by inversion to homogenize. Place pre‐chilled tubes in the cold tube rack to maintain a low temperature while aliquoting. Prepare single‐use aliquots of 60 and 120 µl. Immediately store aliquots at −20°C.

Geltrex must be kept on ice at all times to prevent solidification.

To coat plates or dishes with Geltrex:

1. Thaw an aliquot of Geltrex on ice.
  Thaw Geltrex aliquots only once.
2. Prepare a 1:100 dilution of Geltrex in cold DMEM/F‐12, HEPES (Fisher Scientific, cat. no. 11330057) basal medium. Dispense solution into tissue culture–treated, sterile plates or dishes immediately and incubate for ≥1 hr at 37°C.
  To coat a 6‐well plate, supplement 6 ml cold DMEM/F‐12/HEPES with 60 µl Geltrex. Add 1 ml of this solution to each well.
3. When the plates or dishes are ready, aspirate basal medium and directly add cell suspension.
  It is not necessary to wash the plates/dishes before plating the cells.
  Do not let the Geltrex‐coated plates/dishes dry; aspirate the basal medium just before plating.
4. Use plates or dishes within 24 hr or add DMEM/F‐12, HEPES basal medium to prevent drying.
  Geltrex‐coated plates/dishes can be used after ≤1 week of incubation at 37°C or after ≤2 weeks if stored at 4°C. Before using Geltrex‐coated plates/dishes stored at 4°C, equilibrate them in the incubator for ≥10 min prior to plating.

### MACS buffer

HBSS (with calcium and magnesium and without phenol red; Thermo Fisher, cat. no. 14025092)
1% (v/v) sodium pyruvate (Thermo Fisher, cat. no. 11360070)
1% (v/v) GlutaMAX (Thermo Fisher, cat. no. 35050061)
100 U/ml penicillin‐streptomycin (10,000 U/ml stock; Thermo Fisher, cat. no. 15140122)
1% (v/v) HEPES (Thermo Fisher, cat. no. 15630106)
0.5% (w/v) bovine serum albumin (BSA; Fisher Scientific, cat. no. BP1600‐100)
Filter sterilize using filter bottle (e.g., Millipore, cat. no. SCGVU02RE)
Store ≤1 month at 4°C

### N2 medium

Neurobasal medium (Thermo Fisher, cat. no. 21103‐049)
1% (v/v) N2 supplement (Thermo Fisher, cat. no. 17502048)
2% (v/v) B27 supplement (minus vitamin A; Thermo Fisher, cat. no. 12587010)
100 U/ml penicillin‐streptomycin (10,000 U/ml stock; Thermo Fisher, cat. no. 15140122)
1% (v/v) GlutaMAX (Thermo Fisher, cat. no. 35050061)
Filter sterilize using filter bottle (e.g., Millipore, cat. no. SCGVU02RE)
Store ≤2 weeks at 4°C

### NPC medium

Complete neurobasal medium (see recipe)
20 ng/ml recombinant human FGFb (PeproTech, cat. no. 100‐18C)
20 ng/ml recombinant human EGF (Thermo Fisher, cat. no. PHG0311)
Store small volume ≤1 week at 4°C

### Serum‐free medium

KnockOut DMEM/F‐12 (Thermo Fisher, cat. no. 12660012)
15% (v/v) KnockOut Serum Replacement (Thermo Fisher, cat. no. 10828028)
1% (v/v) GlutaMAX (Thermo Fisher, cat. no. 35050061)
1% (v/v) MEM Non‐Essential Amino Acids (NEAA; Thermo Fisher, cat. no. 11140050)
100 U/ml penicillin‐streptomycin (10,000 U/ml stock; Thermo Fisher, cat. no. 15140122)
0.001% (v/v) beta‐mercaptoethanol (Thermo Fisher, cat. no. 21985023)
Filter sterilize using filter bottle (e.g., Millipore, cat. no. SCGVU02RE)
Store ≤2 weeks at 4°C

## COMMENTARY

### Background Information

Over the past decade, iPSC‐based technologies have demonstrated many advantages to model a wide range of human diseases (Liu, Oikonomopoulos, Sayed, & Wu, 2018). Their unique versatility and ability to retain the genetic background of the patient donor offer to yield new perspectives in disease biology and spark the promise of more translational drug discovery efforts. Mono‐cultures, co‐cultures, and 3D cultures provide many opportunities to study cell‐autonomous and non‐cell‐autonomous mechanisms of disease onset and progression (Gonzalez, Gregory, & Brennand, 2017; Passier, Orlova, & Mummery, 2016). Differentiation of iPSCs into cell types of interest such as dopaminergic neurons and astrocytes is critical to the success of iPSC‐based models. Whereas dual SMAD inhibition has been established as a neural‐induction standard to produce midbrain‐patterned NPCs and dopaminergic neurons (Kriks et al., 2011), differentiation of astrocytes can be achieved through multiple approaches (TCW et al., 2017). The methods described here are highly relevant to the study of Parkinson's disease because they take advantage of the well‐established dual SMAD inhibition strategy to generate neurons and astrocytes from the same regionally patterned NPCs. These neurons and astrocytes can be studied separately, as mono‐cultures, to describe cell‐autonomous effects of Parkinson's disease, or they can be co‐cultured to elucidate non‐cell‐autonomous mechanisms. Importantly, the co‐culture system allows culture of wildtype and mutant cells to characterize the cell type‐specific effects of mutations.

### Critical Parameters

#### Good cell culture practices

It is critical to prevent cross‐contamination of the cells. The risk of accidentally cross‐contaminating plates increases with the number of cell types maintained in an incubator. It is highly recommended to organize the cell culture routine in a systematic manner. For example, process each cell type independently and clean the hood and aspirator between two different cell types. Avoid working with plates of different cell types in the biosafety cabinet simultaneously.

#### Sterility

It is critical to ensure that all steps of the protocols are performed under sterile conditions. Media and buffers need to be filtered through a 0.22‐µm filter. It is highly recommended to prepare aliquots of media and buffers for daily use; this technique prolongs the shelf‐life of large bottles of media and buffers and reduces the risk of contamination.

#### Quality of the iPSCs

The health and quality of the iPSCs (Basic Protocol 1) are critical parameters for the success of all the cell types produced in Basic Protocols 1 to 3. iPSCs must be monitored and the medium replaced daily. To reduce variability and maintain iPSC viability, we suggest culturing iPSCs in a feeder‐free system, on Geltrex‐coated plates, and in mTeSR1 medium. Our laboratory has had success using mTeSR1 Plus (Stemcell Technologies, cat. no. 05825) to reduce the frequency of medium changes and maintain tight, undifferentiated colonies. iPSCs need to be karyotyped regularly (every 2 months) to ensure their suitability for experiments and their karyotype stability. When necessary, differentiated cells in the iPSC culture must be removed manually and under sterile conditions.

#### Quality control of all cell types produced

It is critical to perform quality‐control experiments on all the cell types produced. Depending on the line, the quality control can be done by qPCR or immunofluorescence and should also include functional assays. The Support Protocol, Basic Protocol 2 (step 10e), and the Understanding Results section contain suggestions of the most critical quality‐control experiments to perform.

### Troubleshooting

Please see Table 2 for a troubleshooting guide.

**Table 2:** Troubleshooting Guide for Preparation of iPSC‐Derived Dopaminergic Neurons and Astrocytes

Problem	Possible cause	Solution
Significant cell death	1. Contamination of the culture 2. Cell culture medium or supplements too old 3. Poor quality of the starting cell type (iPSCs or NPCs)	1. Maintain sterility at all times. 2. Use complete culture medium (e.g., complete neurobasal medium, ScienCell astrocyte medium) within3 weeks. Use NPC medium within 1 week. Use dopaminergic neuron differentiation medium within 4 days. To prevent degradation of heat‐sensitive nutrients, only pre‐warm small aliquots of cell culture medium. 3. Try a second differentiation at the next passage, and if the differentiation fails a second time, thaw a new vial of cells. It is very important to monitor cell viability and morphology daily to prevent propagation of poor‐quality cultures.
Cell death around Day 5 of iPSC‐to‐NPC differentiation	1. No formation of a dense monolayer of iPSCs at Day 0 2. Dense culture leading to a lack of nutrients	1. Ensure that the iPSCs form a dense monolayer at Day 0. If there are “holes,” feed the cells with iPSC medium and start differentiation the next day. 2. Increase the volume of cell culture medium added to each well and feed the cells daily.
iPSCs do not form a confluent monolayer at Day 0	1. ROCK inhibitor too old 2. Cell density at plating too low 3. Cells disturbed within 24 hr of plating	1. Thaw a new aliquot of ROCK inhibitor or make fresh stocks. 2. Increase the cell density at plating. 3. Do not disturb the cells within 24 hr of plating.
Astrocytes grow too slowly or too fast	Growth of different lines of iPSCs/NPCs at different paces	Adjust the starting number of wells for differentiation accordingly. For example, astrocyte differentiation of a slow‐growing line may need to be started in three wells of a 6‐well plate instead of the usual one individual well of a 6‐well plate.
Astrocytes do not express genes of interest at the same level	Poor or no quality control of NPCs	Confirm that the NPCs pass quality control (see Support Protocol) and make new astrocytes from the same batch of NPCs. If the NPCs do not pass quality control, make new astrocytes from a different batch of NPCs.
NPC culture fails before producing dopaminergic neurons (due to extensive cell death or lack of differentiation)^a^	1. Unhealthy NPCs 2. Medium or supplements too old or degraded	1. Ensure that the NPCs are healthy. Some lines may not produce a healthy neuronal culture after 7 to 8 passages; however, this must be experimentally established for each NPC line. 2. Ensure that the medium and supplements are not expired or degraded by prolonged heating or storage. If the suggestions above do not help, produce a new NPC line from healthy iPSCs.
Low number of dopaminergic neurons by immunofluorescence	1. Poor or no quality control of NPCs 2. Poor antibody quality 3. Poor quality of the starting NPCs	1. Confirm that the NPCs pass quality control (see Support Protocol). 2. Confirm the quality of the antibody used for immunofluorescence. 3. Try a new differentiation; if the yield is low a second time, this NPC line should be discarded.

a It is normal for a significant number of NPCs to die during the first days of differentiation. The extent of cell death varies between lines. If unsure, maintain the culture for up to 15 days, as it may be a “slow producer” of neurons.

### Understanding Results

The protocols provide detailed instructions to prepare iPSC‐derived cells (Basic Protocols 1 to 3) and co‐cultures (Basic Protocol 4) relevant to the study of neurobiology and neurodegenerative diseases. Basic Protocol 1 describes differentiation of iPSCs into midbrain‐patterned NPCs. Validation of midbrain patterning is critical to ensure the NPCs’ ability to efficiently produce dopaminergic neurons. NPCs self‐renew and can be maintained for several weeks, as long as they are fed daily and passaged weekly. Basic Protocol 2 describes differentiation of NPCs into astrocytes. It is important to be aware that the midbrain patterning of the NPCs may influence the gene expression profile and biological functions of the NPC‐derived astrocytes. Basic Protocol 3 describes differentiation of midbrain‐patterned NPCs into dopaminergic neurons. The differentiation results in a mixture of neurons and undifferentiated NPCs. It is important to remove these NPCs, as they may influence experimental results. The quality of the neuronal culture needs to be assessed by estimating the number of neurons positive for the dopaminergic‐neuron marker tyrosine hydroxylase (TH). A successful culture contains ∼50% TH^+^ neurons a few days after differentiation and ∼70% after several weeks in culture. Early after differentiation, young neurons are immature, and maturation strategies include extended time in culture and treatment with exogenous factors such as progerin (Miller et al., 2013; Sandoe & Eggan, 2013). Neurons co‐cultured with astrocytes develop features of mature neurons, such as increased formation of synapses (Tang et al., 2013). Basic Protocol 4 describes how to set up and maintain a co‐culture of neurons and astrocytes. The most critical parameter in this culture system is to identify culture conditions suitable for both neurons and astrocytes. Most of the factors used to differentiate dopaminergic neurons negatively affect astrocyte viability; therefore, they are omitted from the co‐culture medium. Furthermore, iPSC‐derived astrocytes require supplementation with 0.5% FBS to maintain a healthy culture. Our laboratory routinely maintains co‐cultures for 2 weeks, but they could be maintained for longer periods of time. In 2 weeks, the neurons develop extended networks of neurites.

### Time Considerations

Preparation and maintenance of NPCs (Basic Protocol 1) require 20 min (visual inspection, medium change) to 1 hr (passaging) every day. The protocol from initial plating of the iPSCs to freezing the first passage of NPCs is accomplished in 18 days. The cultures can be maintained for at least seven passages, until they are not able to produce neurons and astrocytes. Cultures should be discarded if the cells display unusual morphology and growth patterns as well as abnormal cell death.

Quality control of the NPCs by qPCR (Support Protocol) requires 1 day, from RNA extraction to data analysis.

Differentiation of NPCs into astrocytes (Basic Protocol 2) is accomplished in 1 month. It requires 20 min (visual inspection, medium change) to 1 hr (passaging) every other day. At the final stage of differentiation (Day 28), freezing astrocytes for cryostorage requires ∼1.5 hr. Depending on the line, the astrocytes can be maintained until passage 5 or later.

Differentiation of NPCs into dopaminergic neurons (Basic Protocol 3) is accomplished in 7 to 10 days. It requires 20 min (visual inspection, medium change) to 2 hr (MACS depletion and plating) per day. The dopaminergic neurons can be maintained for at least 60 days when plated on Geltrex‐coated plates.

NPCs and astrocytes can be cryopreserved and thawed as necessary. These cell types can therefore be prepared in large batches and thawed as needed. Preparation of astrocyte/neuron co‐culture requires ≥3 days: 1 day to plate the astrocytes (40 min of active time), 1 day to treat the astrocytes with AraC (15 min), and 1 day to harvest and plate the dopaminergic neurons (2 hr). In our laboratory, co‐cultures are maintained for 14 days, but they could be maintained longer.
